# A comparison of models with weight, height, and BMI as predictors of mortality

**DOI:** 10.1002/osp4.473

**Published:** 2020-12-17

**Authors:** Kimmo Sorjonen, Gustav Nilsonne, Daniel Falkstedt, Tomas Hemmingsson, Bo Melin, Michael Ingre

**Affiliations:** ^1^ Department of Clinical Neuroscience Karolinska Institutet Stockholm Sweden; ^2^ Department of Psychology Stockholm University Stockholm Sweden; ^3^ Institute of Environmental Medicine Karolinska Institutet Stockholm Sweden; ^4^ Department of Public Health Sciences Stockholm University Stockholm Sweden; ^5^ Institute for Globally Distributed Open Research and Education (IGDORE) Stockholm Sweden

**Keywords:** BMI, conscripts, height, mortality, prediction, weight

## Abstract

**Introduction:**

Body mass index (BMI) is a composite variable of weight and height, often used as a predictor of health outcomes, including mortality. The main purpose of combining weight and height in one variable is to obtain a measure of obesity independent of height. It is however unclear how accurate BMI is as a predictor of mortality compared with models including both weight and height or a weight × height interaction as predictors.

**Methods:**

The current study used conscription data on weight, height, and BMI of Swedish men (*N* = 48,904) in 1969/70 as well as linked data on mortality (3442 deaths) between 1969 and 2008. Cox proportional hazard models including combinations of weight, height, and BMI at conscription as predictors of subsequent all‐cause and cause‐specific mortality were fitted to data.

**Results:**

An increase by one standard deviation on weight and BMI were associated with an increase in hazard for all‐cause mortality by 5.4% and 11.5%, respectively, while an increase by one standard deviation on height was associated with a decrease in hazard for all‐cause mortality by 9.4%. The best‐fitting model indicated lowest predicted all‐cause mortality for those who weighed 60.5 kg at conscription, regardless of height. Further analyses of cause‐specific mortality suggest that this weight seems to be a compromise between lower optimal weights to avoid cancer and CVD mortality and a higher optimal weight to not die by suicide.

**Conclusions:**

According to the present findings, there are several ways to make better use of measured weight and height than to calculate BMI when predicting mortality.

## 1 INTRODUCTION

Body mass index (BMI) has been shown to predict various health outcomes, including cardiometabolic disease,^1^ cancers,^2^, ^3^ and all‐cause mortality.^4^, ^5^, ^6^, ^7^, ^8^ The logic of using BMI rather than body weight as a predictor assumes that health consequences of a person's weight depends on their height, for example that the impact of weighing 100 kg is different for a person who is 200 cm tall than for a person who is 150 cm tall, and that every kilo has greater impact the shorter the person is. By taking a ratio and constructing a composite variable, an attempt is made to attenuate or control for this effect of height. However, this approach introduces assumptions that may be unintended, leads to problems with interpretability, and may reduce statistical power and obscure relationships between height and weight.^9^

By definition, an interaction effect (two‐way) means that the association between one of the predictors (*x*_1_) and the outcome (*y*) depends on the value of the other predictor (*x*_2_).^10^ If, for example, weight and height were to interact in their effect on mortality, this would mean that the association between weight and mortality depends on height and that the association between height and mortality depends on weight.

Using a ratio as a predictor variable in regression modeling is equivalent to modeling an interaction effect without modeling the main effects. This introduces a risk that main effects are interpreted as evidence for interaction effects.^9^, ^11^ If two predictors *x*_1_ and *x*_2_ are not perfectly positively correlated, those with a high value on *x*_1_ (or a low value on *x*_2_) will also tend to have a high value on the *x*_1_ */x*_2_ ratio. Consequently, an association between the *x*_1_ */x*_2_ ratio and the outcome *y* could be due to a simple association between *x*_1_ or *x*_2_ and *y* without any interaction effect.^9^ This problem affects many ratios which have been used as independent variables in research. For instance, Richardson^12^ has criticized interpretations of the waist–hip ratio (WHR) as implying an interaction between waist and hip circumferences, in cases where waist circumference alone is a better predictor.

However, even if main effects of *x*_1_ and *x*_2_ are included in the model, a significant effect of the *x*1 *× x*2 product does not necessarily indicate the presence of an interaction effect. If *x*1 and *x*2 are correlated, the *x*1 *× x*2 product will tend to be correlated with the *x*1^*2*^ and *x*2^*2*^ squared terms. Hence, an identified interaction effect could actually be due to a quadratic association between *y* and *x*1 or *x*2. Therefore, it is necessary to control for such quadratic associations before claiming that *x*_1_ and *x*_2_ interact in their effect on *y*.^13^

This study investigates whether an explicit weight × height interaction effect is a better way to model effects of weight and height on health outcomes, compared with BMI. As discussed above, an analysis with BMI as a predictor but omitting main effects of weight and height is not a proper test of an interaction effect, and considering that the correlation between BMI and weight in adult populations tends to be as strong as 0.9,^14^ the indicated health consequences of BMI could, actually, be due to simple effects of weight. The objective of the present study was, therefore, to analyze the weight × height interaction effect on the ultimate health outcome, mortality, and to compare the predictive power of this interaction effect to that of BMI. But, as weight and height are correlated, a significant effect of the weight × height product, indicating an interaction effect, could be due to a curvilinear effect of either weight or height.^13^ Therefore, also a model including the quadratic weight and height terms, as well as the weight × height interaction, was fitted to data.

## 2 METHOD

### 2.1 Participants and measures

The present study was based on data from 48,904 Swedish males, born between 1949 and 1951. In 1969/70 these men were assessed before their compulsory military service. At this conscript board examination, tests of physical and cognitive capacity, as well as medical check‐ups, including measures of weight and height, were conducted. In the present article, the assessed young men are called “conscripts.” At that time, only 2%–3% of all Swedish men were exempted from conscription, in most cases owing to severe handicaps or congenital disorders.

The conscripts were also asked about smoking and drinking habits and were categorized into smokers (*N* = 28,180) and non‐smokers (*N* = 19,987) and into those with (*N* = 6312) and without (*N* = 34,149) risky alcohol consumption. Alcohol consumption was defined as risky if it exceeded 250 g of pure alcohol per week, if alcohol was used to ease hangover, if the conscript had been apprehended for drunkenness, or if he reported to be drunk often. The conscripts could also be linked to information from the National Population and Housing Census on their father's occupational position in 1960, categorized into manual and non‐manual, when the subjects were between nine and eleven years old.

For information on mortality, the cohort of conscripts was linked to the National Cause of Death Register 1969–2008, held by the National Board of Health and Welfare. The conscripts were followed with regard to all‐cause mortality, and to major cause‐specific mortality: (1) cancer mortality [ICD codes, 8th and 9th (139–209) and 10th (C) revisions], (2) cardiovascular disease (CVD) mortality [ICD codes, 8th and 9th (390–459), and 10th (I00–I99) revisions], (3) alcohol‐related mortality [ICD codes, 8th and 9th (291, 303) and 10th (F10, K70, K74) revisions], (4) intentional injuries (suicides) [ICD codes, 8th and 9th (950–959 + 980–989) and 10th (X60–X84 + Y12–Y34) revisions], and (5) unintentional injuries [ICD codes, 8th and 9th (800–999) and 10th (V–Y) revisions, except suicides (950–959 + 980–989/X60–X84 + Y12–Y34)].

### 2.2 Ethics

The Stockholm Regional Ethical Review Board has in decisions according to minutes 2004/5:9 agreed to co‐processing of the compulsory military service material. The inclusion of more recent data to the database has also been approved by the Review Board (Dnr 2008/323‐32 and 2010/604‐32). Only non‐identifying information was available for the present study. Due to the character of the database and the anonymization of all data, the Review Board waived the requirement for written consent.

### 2.3 Statistical analyses

Weight, height, and BMI were standardized before calculating the quadratic and interaction terms and inclusion as predictors in the models (except Model 5). Using Cox proportional hazard regression, the association between hazard for all‐cause and cause‐specific mortality and predictors were analyzed in nine different models: (1–3) Univariate association with weight, height, and BMI; (4) Curvilinear association with BMI; (5) Association with BMI categorized as underweight (<18.5), normal weight (18.5–25, reference), overweight (25–30), and obesity (>30); (6) Multivariate association with weight and height; (7) Association with weight, height, and the weight × height interaction; (8) As BMI = weight (kg)/height (m)^2^ = weight (kg) × height (m)^−2^, it is possible that the health impact of weight is assumed to be a function of height^−2^. Therefore, also a model with weight, height^−2^, and weight × height^−2^ as predictors was analyzed; and (9) Association with weight, height, their interaction, as well as quadratic terms.

Model fit was evaluated by Akaike information criterion (AIC), for which a lower value indicates better fit. AIC takes the number of predictors into consideration and rewards, with a lower value, parsimonious models with few predictors.^15^ Also, the differences between predicted hazard for mortality between the nine models were used as predictors of mortality. Here, a significant effect indicates that one of the models is making better predictions of the actual observed hazard. Analyses were conducted with R 4.0.2 statistical software^16^ employing the survival package.^17^ Script and data are available at https://osf.io/82cpq/.

## 3 RESULTS

Descriptive statistics for, and correlations between, study variables are presented in Table 1. A high correlation between BMI and weight can be noted. In Table 2 fit measures and parameter values for the nine models (see Section 2) are presented.

**TABLE 1:** Descriptive statistics for, and correlations between, study variables

Variable	1	2	3	4
1. Weight	–	0.472**	0.856**	0.014*
2. Height	–	–	−0.047**	−0.026**
3. BMI	–	–	–	0.031**
4. Deceased^a^	–	–	–	–

*N*	48,904	48,904	48,904	49,321
*M*	66.64	178.19	20.97	0.07
*SD*	9.26	6.36	2.57	–

^a^ 0 for alive and 1 for deceased.

*p < 0.005, **p < 0.001.

**TABLE 2:** Number of deaths as well as model fit (AIC) and parameter values (hazard ratios, with 95% CI) for nine evaluated models for six different causes of death

Model/Par.	All‐cause	Cancer	CVD	Alcohol	Suicide	Injuries
Deaths	3442	840	676	205	641	575
Model 1	74,073	**18,046**	14,494	4410	13,799	12,391
Weight	1.054 (1.020; 1.089)*	1.169 (1.098; 1.245)**	1.270 (1.189; 1.357)**	0.943 (0.818; 1.086)	0.853 (0.785; 0.927)**	0.956 (0.879; 1.040)
Model 2	74,049	18,063	14,539	**4397**	13,781	**12,377**
Height	0.906 (0.876; 0.937)**	1.081 (1.011; 1.157)^†^	0.977 (0.906; 1.054)	0.773 (0.674; 0.887)**	0.795 (0.736; 0.859)**	0.849 (0.782; 0.921)**
Model 3	74,038	18,052	14,478	4409	13,813	12,391
BMI	1.115 (1.081; 1.151)**	1.139 (1.070; 1.213)**	1.307 (1.227; 1.391)**	1.085 (0.952; 1.237)	0.956 (0.882; 1.035)	1.045 (0.965; 1.132)
Model 4	74,019	18,052	**14,476**	4409	13,814	12,392
BMI	1.056 (1.017; 1.097)*	1.099 (1.017; 1.188)^†^	1.228 (1.128; 1.336)**	1.019 (0.874; 1.188)	0.936 (0.856; 1.023)	1.018 (0.927; 1.119)
BMI^2^	1.030 (1.018; 1.042)**	1.020 (0.995; 1.045)	1.024 (1.003; 1.046)^†^	1.036 (0.991; 1.084)	1.019 (0.982; 1.058)	1.018 (0.983; 1.054)
Model 5	74,032	18,056	14,485	4413	13,817	12,395
Underw.^a^	0.998 (0.904; 1.101)	1.023 (0.839; 1.249)	0.881 (0.694; 1.120)	0.928 (0.613; 1.404)	0.961 (0.765; 1.208)	0.957 (0.751; 1.220)
Overw.^a^	1.346 (1.184; 1.531)**	1.490 (1.161; 1.914)*	2.041 (1.597; 2.609)**	1.220 (0.707; 2.106)	0.866 (0.606; 1.238)	1.007 (0.707; 1.434)
Obese^a^	2.436 (1.894; 3.134)**	2.302 (1.356; 3.909)*	4.410 (2.850; 6.824)**	1.962 (0.626; 6.148)	1.194 (0.534; 2.670)	1.569 (0.744; 3.311)
Model 6	74,008	18,048	14,480	4398	13,781	12,378
Weight	1.131 (1.091; 1.172)**	1.167 (1.088; 1.252)**	1.355 (1.262; 1.455)**	1.085 (0.932; 1.263)	0.946 (0.863; 1.037)	1.044 (0.952; 1.144)
Height	0.854 (0.823; 0.887)**	1.004 (0.931; 1.083)	0.843 (0.775; 0.916)**	0.744 (0.637; 0.869)**	0.816 (0.747; 0.891)**	0.832 (0.758; 0.913)**
Model 7	74,001	18,048	14,482	4400	13,776	12,380
Weight	1.128 (1.088; 1.169)**	1.158 (1.078; 1.244)**	1.352 (1.259; 1.453)**	1.085 (0.931; 1.264)	0.951 (0.868; 1.041)	1.043 (0.951; 1.143)
Height	0.855 (0.823; 0.887)**	1.000 (0.927; 1.079)	0.840 (0.772; 0.914)**	0.744 (0.637; 0.869)**	0.833 (0.761; 0.911)**	0.831 (0.757; 0.911)**
W × H	1.042 (1.015; 1.070)*	1.031 (0.978; 1.087)	1.014 (0.959; 1.072)	0.997 (0.886; 1.123)	1.092 (1.029; 1.159)*	0.983 (0.913; 1.057)
Model 8	74,001	18,048	14,481	4400	13,778	12,380
Weight	1.128 (1.089; 1.170)**	1.157 (1.077; 1.244)**	1.355 (1.261; 1.455)**	1.080 (0.927; 1.258)	0.948 (0.865; 1.039)	1.039 (0.948; 1.139)
Height^−2^	1.166 (1.124; 1.210)**	0.998 (0.925; 1.078)	1.192 (1.097; 1.294)**	1.333 (1.147; 1.549)**	1.188 (1.087; 1.298)**	1.196 (1.092; 1.311)**
W × H^−2^	0.967 (0.942; 0.992)^†^	0.965 (0.915; 1.019)	0.991 (0.937; 1.049)	1.019 (0.908; 1.143)	0.933 (0.881; 0.989)^†^	1.028 (0.957; 1.106)
Model 9	**73,978**	18,046	14,477	4399	**13,775**	12,379
Weight	1.056 (1.013; 1.101)^†^	1.110 (1.017; 1.212)^†^	1.260 (1.146; 1.386)**	1.029 (0.869; 1.218)	0.896 (0.814; 0.985)^†^	1.022 (0.921; 1.133)
Height	0.876 (0.843; 0.911)**	1.020 (0.944; 1.103)	0.862 (0.791; 0.939)**	0.761 (0.650; 0.892)**	0.839 (0.766; 0.918)**	0.839 (0.763; 0.923)**
Weight^2^	1.042 (1.028; 1.055)**	1.027 (0.999; 1.055)	1.031 (1.004; 1.057)^†^	1.040 (0.982; 1.101)	1.055 (1.020; 1.091)*	1.018 (0.978; 1.060)

*Note:* The parameter values give the predicted multiplicative change in the hazard for mortality for an increase in the predictor by one standard deviation. For example, an increase in height by one *SD* is associated with a decrease in the hazard for all‐cause mortality with 9.4%. In each column, the lowest AIC (= the best fit, rewarding parsimoniousness) is given in bold.

^a^ Reference = normal weight.

^†^ *p* < 0.05, *p < 0.01, **p < 0.001.

### 3.1 All‐cause mortality

As seen in Table 2, based on AIC, BMI is a more accurate univariate predictor of all‐cause mortality than weight and height taken separately (Model 3 vs. Models 1 and 2). The model including the quadratic term of BMI (Model 4) is better still, indicating a nadir at BMI = 18.6 kg/m^2^. The quadratic effect of BMI is also better at predicting all‐cause mortality than categorized BMI (Model 5). However, this quadratic model makes less accurate predictions than Model 6 including both weight and height as predictors. Even more accurate predictions of all‐cause mortality are given by the two models (7 and 8) including interaction terms, and of these two Model 7 is probably preferable due to simplicity and interpretability of parameter values. However, the best fit was exhibited by Model 9 with weight, height, and the quadratic term of weight as predictors (adding a quadratic term of height or the weight × height interaction to this model did not improve it significantly). According to Model 9, the hazard for all‐cause mortality decreases with height, and for every height the lowest hazard is predicted for those with standardized weight = −0.665, which corresponds to a weight of 60.5 kg. As seen in Table 3, Model 9 is making significantly better predictions of all‐cause mortality compared with the other eight models.

**TABLE 3:** All pairwise comparisons of the predictions made by the nine models of the six causes of mortality

Denominator	All‐cause (above diagonal) and cancer (below diagonal) mortality								
	Numerator model								
	1	2	3	4	5	6	7	8	9
1	–	1.49*	6.70**	3.90**	3.49**	2.72**	2.68**	2.68**	2.77**
2	2.67**	–	1.15	1.39*	1.26^†^	2.70**	2.59**	2.61**	2.64**
3	3.32*	0.61^†^	–	2.65**	1.27	2.90**	2.63**	2.66**	2.63**
4	2.26^†^	0.57*	0.48	–	0.47*	1.52*	1.66**	1.67**	2.70**
5	1.63	0.60^†^	1.09	1.41	–	1.57**	1.67**	1.68**	2.60**
6	0.09	0.37**	0.33*	0.46^†^	0.62	–	3.07**	2.96*	2.85**
7	0.48	0.36**	0.34*	0.45^†^	0.60^†^	0.50	–	0.43	2.46**
8	0.47	0.36**	0.34*	0.45^†^	0.59^†^	0.48	0.00	–	2.38**
9	0.50	0.36**	0.36*	0.32*	0.46*	0.50	0.62	0.75	–

Denominator	CVD (above diagonal) and alcohol related (below diagonal) mortality								
	Numerator model								
	1	2	3	4	5	6	7	8	9
1	–	0.40**	2.89**	2.81**	1.33	2.53**	2.43**	2.44**	2.29**
2	0.32**	–	2.71**	2.71**	2.71**	2.71**	2.71**	2.72**	2.71**
3	0.82	2.31*	–	0.79	0.72	2.47	1.78	2.98	1.20
4	0.62	2.07*	0.35	–	0.62	1.39	1.40	1.69	1.82
5	0.76	2.28*	0.72	3.60	–	1.39	1.40	1.43	1.64^†^
6	0.37**	0.38	0.37**	0.44*	0.39**	–	3.52	12.44	1.14
7	0.37**	0.37	0.37**	0.44*	0.39**	0.00	–	558.50^†^	1.06
8	0.37**	0.53	0.37**	0.44*	0.39**	151.62	300.26	–	0.80
9	0.38**	0.41	0.37**	0.39**	0.37**	0.36	0.38	0.35	–

Denominator	Suicide (above diagonal) and injuries (below diagonal) mortality								
	Numerator model								
	1	2	3	4	5	6	7	8	9
1	–	1.92**	0.21**	0.24**	0.39**	2.61**	2.61**	2.66**	2.31**
2	0.32**	–	0.40**	0.42**	0.39**	2.70	2.27^†^	1.86	2.58*
3	1.00	2.42**	–	2.45	0.84	2.68**	2.74**	2.74**	2.69**
4	0.84	2.26**	0.36	–	0.66	2.54**	2.65**	2.64**	2.91**
5	0.95	2.40**	0.85	2.04	–	2.60**	2.63**	2.63**	2.62**
6	0.37**	0.37	0.37**	0.40**	0.39**	–	1.94^†^	1.58	2.25*
7	0.37**	0.31	0.36**	0.40**	0.39**	0.26	–	0.00**	1.26
8	0.37**	0.78	0.37**	0.40**	0.39**	4.11	8975.66*	–	1.81
9	0.37**	0.40	0.37**	0.38**	0.38**	0.38	0.77	0.47	–

*Note:* Values above one indicates that the model in the numerator (column) is better at predicting mortality while values below one indicates that the model in the denominator (row) is better.

^†^ *p* < 0.05, *p < 0.01, **p < 0.001.

**FIGURE 1:**
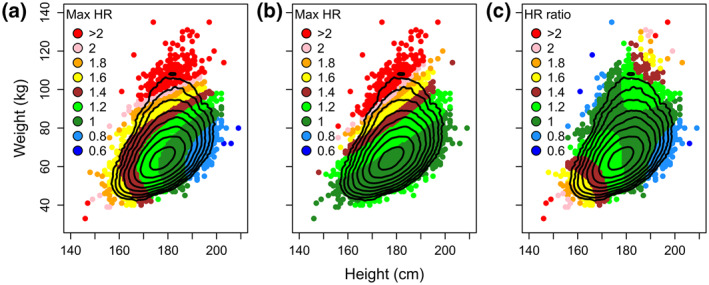
Association between height and weight, with each contour indicating a doubling of density of observations. The colors show predicted hazard for mortality given by the model with weight, height, and weight^2^ as predictors (Model 9, panel A) and the model with BMI and BMI^2^ as predictors (Model 4, panel B). Panel C shows the ratio of the predicted hazard from Model 9 divided by the predicted hazard from Model 4

The model with weight, height, and the quadratic term of weight (Model 9) was evaluated further by comparing it with the most accurate BMI model including a quadratic term of BMI (Model 4). The density of observations for different combinations of height and weight as well as predicted hazard for mortality made by Model 9, Model 4, as well as the ratio of predictions made by these two models, are presented in Figure 1. Compared with Model 4, Model 9 predicts higher hazard for small (short and light) and for large (tall and heavy) men while it predicts lower hazard for tall and light (low BMI) and, to some degree, relatively short and heavy (high BMI) men.

**FIGURE 2:**
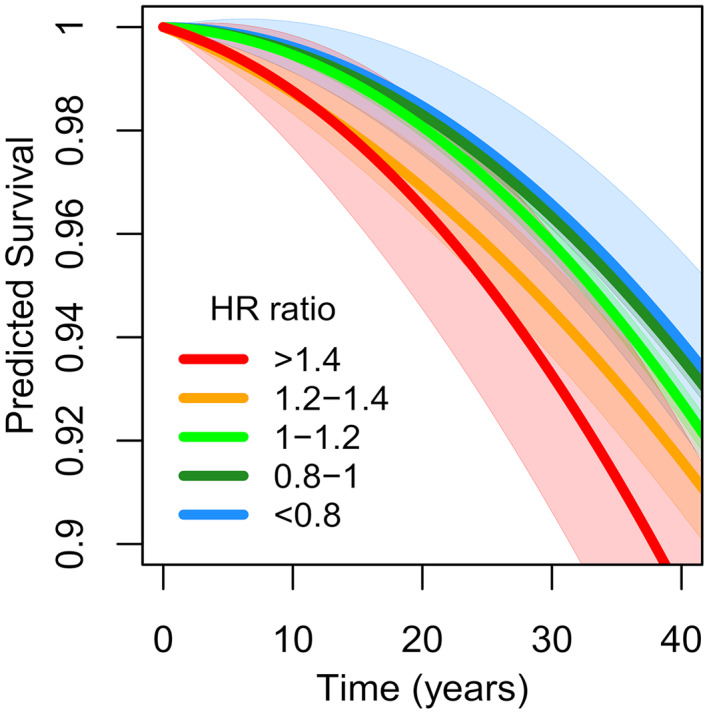
Predicted cumulative survival (95% CI in transparent colors with thin borders) as a function of time since conscription, separately for five levels of the predicted hazard from the height plus quadratic weight model (Model 9) divided by the predicted hazard from the quadratic BMI model (Model 4, see panel C in Figure 1)

The difference between predictions (logged) made by Model 9 and Model 4 was, in turn, used as a predictor of the hazard for all‐cause mortality. An increase by one on this difference (which corresponds to a *e* ^1^ = 2.7‐fold increase in predicted hazard, i.e., approximately corresponding to the ratio between 2 (pink dots) and 0.8 (light blue dots) in Panel C in Figure 1) is associated with a 2.7‐fold increase in the observed hazard (*p* < 0.001). This indicates that the hazard for all‐cause mortality is high if it is predicted to be high by Model 9 compared with Model 4 (Figure 2). For example, small men, for example those who are shorter or equal to 170 cm tall and weigh less or equal to 60 kg (*N* = 3408; 282 deaths), are predicted to have an increased hazard for all‐cause mortality according to Model 9 (mean predicted hazard = 1.22) but not according to Model 4 (mean predicted hazard = 0.96). An analysis comparing these men with the rest confirmed the prediction made by Model 9 (*HR* = 1.21, *p* = 0.003).

In order to make a straightforward comparison of the models, the analyses above were conducted without adjustment for possible confounders. However, if adjusting for smoking and risky alcohol consumption at the time of conscription as well as father's occupational position at age 9–11, the predictive advantage of Model 9 compared with Model 4 increased. Now an increase by one on the difference between predictions (logged) was associated with a 3.5‐fold increase in the observed hazard (*p* < 0.001). When adjusting for these three possible confounders, the nadir for BMI in Model 4 increased to 18.9 kg/m^2^ and for weight in Model 9 to 62.6 kg. Among smokers, the nadirs for BMI and for weight were 17.5 kg/m^2^ and 59.1 kg, respectively. Among non‐smokers, on the other hand, the squared BMI term was not significant and the analysis indicated a positive linear association while the nadir for weight in Model 9 was 49.2 kg, that is, the analysis indicated a positive exponential association.

### 3.2 Cause‐specific mortality

Table 3 shows that with a single exception (Model 8 for CVD mortality), Model 9 is making the best predictions of all cause‐specific deaths, although the difference is not always significant. Model 4, on the other hand, is always making worse predictions than Models 6‐9, and except for CVD mortality significantly so. If parsimoniousness is considered as well as predictive accuracy, AIC (Table 2) indicates that high weight could be seen as the best predictor of cancer mortality, while short stature is the best predictor of mortality due to alcohol and injuries. For CVD mortality, Model 4 predicts a nadir at BMI = 9.9 kg/m^2^ and Model 9 a nadir at weight = 31.0 kg, that is, they both make positive exponential predictions. For suicide Model 9 predicts a nadir at weight = 76.1 kg.

If adjusting for smoking, risky alcohol consumption, and father's occupational position, Model 9 is still making significantly better predictions of deaths due to alcohol, suicide, and injuries compared with Model 4 (all *p*s ≤ 0.001). However, Model 9 stops being a better predictor of cancer mortality (*p* = 0.674) but instead becomes a better predictor of CVD mortality (*p* < 0.001).

## 4 DISCUSSION

The present study tested whether BMI is the best way to use weight and height to predict mortality. Results show that regression models using BMI as an independent variable are in general inferior to models where height and weight are entered separately as independent variables. This finding highlights drawbacks of using ratio composite variables in regression modeling.

Previous studies have concluded that for every height there is an optimal, but varying, weight that results in an optimally survival enhancing BMI.^4^, ^5^, ^6^, ^7^, ^8^ Based on data from the present cohort of young Swedish male conscripts, the optimal weight would be the one that results in a BMI of 18.6 kg/m^2^, although higher optimal values in the range of 20–24,^5^ 20–25,^6^ 22.5–25,^7^ and 22.6–27.5^8^ have been indicated before. However, according to the present findings, there are several ways to make better use of measured weight and height than to calculate BMI when predicting mortality, for example, by simply using weight and height as simultaneous predictors of mortality. As a heuristic, the survival of people, or at least young men, is enhanced by as low weight as possible and high stature.

One exception to this heuristic is the positive association between height and cancer mortality. This association has been observed before^18^, ^19^, ^20^, ^21^ and could be due to taller people having more cells in their body, consistent with the multistage model of carcinogenesis.^22^ Another contributing reason for this positive association could be the negative associations height has with other causes of mortality. As tall men, like everybody else, have to die of something, they might experience an increased hazard for cancer mortality because they have a lower hazard to die of other causes. This interpretation receives support from the fact that the association between height and cancer mortality ceases to be significant when adjusting for weight, thereby indirectly adjusting for, to some degree, the hazard for other causes of death.

An exception to the heuristic that it is advantageous to weigh as little as possible is the negative association between weight and hazard for suicide. This is in accordance with previous studies, that have found a negative prospective association between BMI and suicide,^23^, ^24^, ^25^, ^26^ although the present findings indicate that weight is a better predictor of suicide than BMI. This is probably due to weight being more confounded by height (Table 1), which is a strong predictor of suicide. This interpretation is supported by the fact that weight stops being a significant predictor of suicide when adjusting for height (Table 2). However, even when adjusting for height, weight seems to a have a quadratic prospective association with suicide (Table 2), and the hazard was predicted to be lowest at a weight of 76.1 kg. This does not, of course, necessarily mean that low or excessive weight has a causal effect on the risk for suicide. Low or excessive weight might, instead, be indicative of various mental health problems, although low BMI is more likely to result from a medical than mental health issue.

In the present study, the most accurate model predicted that all‐cause mortality decreases with height and that for every height mortality is lowest for those who weigh 60.5 kg. This weight seems to be some kind of compromise between lower predicted optimal weights to avoid cancer and CVD mortality, and a higher predicted optimal weight to not die by suicide. It is interesting to note that this model with a linear effect of height and a quadratic effect of weight was better than the model with a quadratic effect of BMI at predicting both all‐cause and all of the specific causes of death, although the difference was not significant for CVD mortality.

This study is limited to men who had their weight and height measured at age 18–21 years and who were followed up to age 57–59 years. The observed associations might not apply to women or to men measured at other ages. For example, it is possible that the optimal weight of 60.5 kg and BMI of 18.6 kg/m^2^ would not be observed had the weight and height of the men been measured at a later age, for example 50, when a BMI as low as 18.6 should be quite uncommon and may be indicating a pathological state. This is one possible reason why the optimal BMI in the present study seems to be lower compared with previous studies mentioned above, where participants tended to be considerably older at baseline. It is also possible, even probable, that the results would look different had the follow up period extended into older age. In the present data, a large percentage of total mortality is still due to suicide and injuries, resulting in a relatively strong association between height and all‐cause mortality. However, as the men get older and the relative frequency of cancer and CVD mortality increases, weight, and consequently BMI, can be expected to increase in importance as predictors of all‐cause mortality in the present dataset. Furthermore, as the optimal youth weight is lower for cancer and CVD mortality compared with mortality due to suicide and injuries, the optimal youth weight for all‐cause mortality could be expected to decrease from 60.5 kg, observed in the present study, as the men get older.

## 5 CONCLUSIONS

The present findings suggest that BMI is not an optimal way to model effects of weight and height on mortality. In the present sample, a model with a linear effect of height and a quadratic effect of weight is better at predicting mortality, both all‐cause and cause‐specific, compared with BMI. For all‐cause mortality, a nadir was predicted for a weight of 60.5 kg, which seems to be a compromise between lower optimal weights to avoid cancer and CVD mortality and a higher optimal weight to not die by suicide. The optimal survival enhancing weight might be a function both of age at measurement and length of follow‐up period.

## CONFLICT OF INTEREST

None declared.

## AUTHOR CONTRIBUTIONS

Conceived of the original idea: Kimmo Sorjonen, Gustav Nilsonne, and Michael Ingre. Provided data: Daniel Falkstedt, Tomas Hemmingsson, and Bo Melin. Conducted the statistical analyses: Kimmo Sorjonen and Michael Ingre. Kimmo Sorjonen wrote the script with assistance from Michael Ingre. Wrote an initial draft: Kimmo Sorjonen. All authors discussed the results, proposed changes, and contributed to the final manuscript; all authors have approved the final version of the manuscript.

## References

[1] Lyall DM , Celis‐Morales C , Ward J , et al. Association of body mass index with cardiometabolic disease in the UK biobank: a mendelian randomization study. JAMA Cardiol 2017;2(8):882‐889. 10.1001/jamacardio.2016.5804 28678979 PMC5710596

[2] Bhaskaran K , Douglas I , Forbes H , dos‐Santos‐Silva I , Leon DA , Smeeth L . Body‐mass index and risk of 22 specific cancers: a population‐based cohort study of 5·24 million UK adults. Lancet. 2014;384(9945):755‐765. 10.1016/S0140-6736(14)60892-8 25129328 PMC4151483

[3] Renehan AG , Tyson M , Egger M , Heller RF , Zwahlen M . Body‐mass index and incidence of cancer: a systematic review and meta‐analysis of prospective observational studies. Lancet. 2008;371:569‐578. 18280327 10.1016/S0140-6736(08)60269-X

[4] Adams KF , Schatzkin A , Harris TB , et al. Overweight, obesity, and mortality in a large prospective cohort of persons 50 to 71 years old. N Engl J Med. 2006;355(8):763‐778. 16926275 10.1056/NEJMoa055643

[5] Aune D , Sen A , Prasad M , et al. BMI and all cause mortality: systematic review and non‐linear dose‐response meta‐analysis of 230 cohort studies with 3.74 million deaths among 30.3 million participants. BMJ. 2016;353:i2156. 10.1136/bmj.i2156 27146380 PMC4856854

[6] Berrington de Gonzalez A , Hartge P , Cerhan JR , et al. Body‐mass index and mortality among 1.46 million white adults. N Engl J Med. 2010;363(23):2211‐2219. 10.1056/NEJMoa1000367 21121834 PMC3066051

[7] Prospective Studies Collaboration . Body‐mass index and cause‐specific mortality in 900 000 adults: collaborative analyses of 57 prospective studies. Lancet. 2009;373:1083‐1096. 19299006 10.1016/S0140-6736(09)60318-4 PMC2662372

[8] Zheng W , McLerran DF , Rolland B , et al. Association between body‐mass index and risk of death in more than 1 million asians. N Engl J Med. 2011;364(8):719‐729. 10.1056/NEJMoa1010679 21345101 PMC4008249

[9] Kronmal RA . Spurious correlation and the fallacy of the ratio standard revisited. J R Stat Soc Ser A Stat Soc. 1993;156(3):379‐392. 10.2307/2983064

[10] Jaccard J , Turrisi R . Interaction Effects in Multiple Regression. 2nd ed. Thousand Oaks, CA: Sage Publications; 2003.

[11] Brambor T , Clark WR , Golder M . Understanding interaction models: Improving empirical analyses. Polit Anal. 2006;14(1):63‐82. 10.1093/pan/mpi014

[12] Richardson T . Ratios in the evolutionary behavioural sciences: problems and solutions. PsyArXiv; 2020. 10.31234/osf.io/j7vem

[13] Ganzach Y . Misleading interaction and curvilinear terms. Psychol Methods. 1997;2(3):235‐247.

[14] Diverse Populations Collaborative Group . Weight‐height relationships and body mass index: some observations from the diverse populations collaboration. Am J Phys Anthropol. 2005;128(1):220‐229. 10.1002/ajpa.20107 15761809

[15] Akaike H . A new look at the statistical model identification. IEEE Trans Autom Control;19(6):716‐723.

[16] R Core Team : A Language and Environment for Statistical Computing. R Foundation for Statistical Computing, Vienna, Austria. URL https://www.R-project.org/. Published online 2020.

[17] Therneau T . A Package for Survival Analysis in R. R Package Version 3.1‐12. URL. https://CRAN.R-project.org/package=survival

[18] Davey Smith G , Hart C , Upton M , et al. Height and risk of death among men and women: aetiological implications of associations with cardiorespiratory disease and cancer mortality. J Epidemiol Community Health. 2000;54(2):97‐103. 10.1136/jech.54.2.97 10715741 PMC1731616

[19] Ihira H , Sawada N , Iwasaki M , et al. Adult height and all‐cause and cause‐specific mortality in the Japan Public Health Center‐based prospective study (JPHC). PLoS One 13; 2018:e0197164. 10.1371/journal.pone.0197164 29758048 PMC5951564

[20] The Emerging Risk Factors Collaboration . Adult height and the risk of cause‐specific death and vascular morbidity in 1 million people: individual participant meta‐analysis. Int J Epidemiol. 2012;41(5):1419‐1433. 10.1093/ije/dys086 22825588 PMC3465767

[21] Wirén S , Häggström C , Ulmer H , et al. Pooled cohort study on height and risk of cancer and cancer death. Cancer Causes Control. 2014;25(2):151‐159. 10.1007/s10552-013-0317-7 24173535 PMC3929024

[22] Nunney L . Size matters: height, cell number and a person's risk of cancer. Proc R Soc B Biol Sci. 2018;285(1889):20181743. 10.1098/rspb.2018.1743 PMC6234890 30355711

[23] Amiri S , Behnezhad S . Body mass index and risk of suicide: a systematic review and meta‐analysis. J Affect Disord. 2018;238:615‐625. 10.1016/j.jad.2018.05.028 29957479

[24] Geulayov G , Ferrey A , Hawton K , et al. Body mass index in midlife and risk of attempted suicide and suicide: prospective study of 1 million UK women. Psychol Med. 2019;49(13):2279‐2286. 10.1017/S0033291718003239 30488817 PMC6754262

[25] Magnusson PKE , Rasmussen F , Lawlor DA , Tynelius P , Gunnell D . Association of body mass index with suicide mortality: a prospective cohort study of more than one million men. Am J Epidemiol. 2006;163(1):1‐8. 10.1093/aje/kwj002 16269577

[26] Perera S , Eisen RB , Dennis BB , et al. Body mass index is an important predictor for suicide: results from a systematic review and meta‐analysis. Suicide Life Threat Behav. 2016;46(6):697‐736. 10.1111/sltb.12244 27094229

